# Ambient Air Pollution and Asthma-Coded Hospital Admission Rates in Children Aged 0–3 Years in Spain: A Municipality-Year Urban–Rural Ecological Study

**DOI:** 10.3390/epidemiologia7040097

**Published:** 2026-07-09

**Authors:** Laura Sánchez de Prada, Daniel Vélez-Serrano, Alejandro Alvaro-Meca

**Affiliations:** 1 Departamento de Medicina, Facultad de Medicina, Universidad de Valladolid, 47003 Valladolid, Spain; lsanchezd@saludcastillayleon.es; 2 Departamento de Estadística e Investigación Operativa, Facultad de Matemáticas, Universidad Complutense de Madrid, 28040 Madrid, Spain; danielvelezserrano@mat.ucm.es; 3 Departamento de Medicina Preventiva y Salud Pública, Facultad de Ciencias de la Salud, Universidad Rey Juan Carlos, 28922 Alcorcón, Spain; 4 Centro de Investigación Biomédica en Red de Enfermedades Infecciosas (CIBERINFEC), Instituto de Salud Carlos III, 28029 Madrid, Spain

**Keywords:** asthma, children, hospital admissions, particulate matter, PM_2.5_, PM_10_, air pollution, ecological study, urban–rural differences, Spain

## Abstract

**Background/Objectives:** Asthma-coded hospital admissions in very young children represent a clinically relevant but diagnostically complex marker of severe wheezing/asthma-like morbidity. **Methods:** We conducted a nationwide ecological municipality-year study of children aged 0–3 years in Spain during 2020–2022 using hospital discharge records from the Spanish Minimum Basic Data Set (MBDS/RAE-CMBD). Exposure and outcome were analyzed at the municipality-year level: the exposure metric was the contemporaneous annual mean municipality-level concentration of PM_2.5_, PM_10_, NO_2_, SO_2_, O_3_, and CO, and the outcome was the annual number of asthma-coded admissions, with the population aged 0–3 years as offset. **Results:** The regression panel comprised 20,746 municipality-year observations, 7251 municipalities, and 3231 admissions; 93.7% of municipality-years had zero admissions. In unipollutant Poisson models adjusted for calendar year, rural–urban status, temperature, and relative humidity, incidence rate ratios per interquartile-range increase were 1.54 (95% CI 1.28–1.85) for PM_2.5_ and 1.89 (95% CI 1.59–2.26) for PM_10_. Negative-binomial and province-fixed-effect sensitivity models attenuated but did not remove the particulate-matter signal. The multipollutant model was unstable because of strong collinearity between particulate fractions and was interpreted only as an exploratory sensitivity analysis. **Conclusions:** These findings should be interpreted as ecological, population-level associations between annual ambient particulate pollution and pediatric respiratory hospital burden, not as individual-level causal risk estimates.

## 1. Introduction

Asthma is one of the most common chronic respiratory diseases worldwide and remains a major cause of morbidity in children. In 2019, asthma affected an estimated 262 million people and caused approximately 455,000 deaths globally [1]. Although asthma occurs across all age groups, its clinical and public health relevance is particularly pronounced in childhood, where acute exacerbations may lead to emergency care, hospital admission, school absenteeism, and substantial family and healthcare burden [2]. The distribution of asthma prevalence, severity, and mortality varies substantially across regions, reflecting differences in environmental exposures, healthcare access, socioeconomic conditions, and disease management [3]. Over recent decades, the increasing prevalence of asthma and wheezing disorders in children has reinforced the need to better characterize modifiable environmental determinants of severe respiratory morbidity [4].

Air pollution is among the most relevant environmental exposures for respiratory health. It consists of a complex and spatially heterogeneous mixture of gaseous pollutants and particulate matter, arising from traffic, combustion processes, industrial activity, secondary atmospheric reactions, mineral dust, biomass burning, and other natural and anthropogenic sources [5,6]. Particulate matter is of particular interest in pediatric respiratory epidemiology because particles can reach different compartments of the respiratory tract depending on their aerodynamic diameter. Fine particulate matter, PM_2.5_, can penetrate deeply into the bronchioles and alveolar region, whereas PM_10_ also captures coarser inhalable particles that may contribute to upper and lower airway irritation, inflammation, and oxidative stress. The 2021 World Health Organization air quality guidelines recommend annual mean concentrations not exceeding 5 μg/m^3^ for PM_2.5_ and 15 μg/m^3^ for PM_10_, highlighting the importance of reducing long-term population exposure to particulate pollution [7].

The biological plausibility of an association between particulate air pollution and asthma morbidity is supported by several mechanisms. Inhaled particles may induce airway epithelial injury, oxidative stress, inflammatory cytokine release, immune dysregulation, and increased airway hyperresponsiveness [5,8]. In early life, these mechanisms may be especially relevant because the respiratory and immune systems are still developing, children inhale a greater volume of air relative to body weight than adults, and early-life environmental injury may affect subsequent lung growth and respiratory trajectories [9,10,11]. Exposure to air pollution during pregnancy and early childhood has been linked to altered immune development, epigenetic changes, impaired lung growth, increased susceptibility to wheezing and asthma-like disease, and childhood asthma incidence [12,13,14,15]. Long-term lung-function decline is also clinically relevant in asthma management, even though this broader literature is not specific to air-pollution exposure [16]. However, in children aged 0–3 years, asthma diagnosis is clinically challenging because recurrent wheezing, bronchiolitis, viral lower respiratory infections, and asthma-like episodes may overlap. International asthma guidance emphasizes that recurrent wheeze is common in children aged 5 years or younger and that diagnosis in this age group requires careful clinical interpretation [17]. For this reason, the present study focuses on asthma-coded hospital admissions, defined according to the principal discharge diagnosis recorded in the Spanish Minimum Basic Data Set (MBDS/RAE-CMBD), rather than on confirmed incident asthma.

Previous epidemiological studies have linked short-term air pollution exposure to asthma exacerbations, emergency department visits, and hospital admissions, while long-term exposure has been associated with asthma development and impaired lung function in children [11,18,19,20]. Nevertheless, evidence remains less consistent for very young children and for severe outcomes requiring hospital admission. Interpretation is further complicated by the fact that PM_2.5_, PM_10_, nitrogen dioxide, sulfur dioxide, ozone, and carbon monoxide often coexist as part of a broader ambient pollution mixture. In particular, PM_2.5_ and PM_10_ may be strongly spatially correlated, making it difficult to attribute observed associations to a single particulate fraction. From a public health perspective, however, identifying a robust particulate-matter signal associated with severe pediatric respiratory morbidity may still be informative for air quality policy and prevention strategies.

Spain provides a relevant setting to study this question because it combines densely populated urban areas, rural municipalities with lower traffic-related pollution, marked geographical variability in climate and topography, and nationwide hospital discharge data with high population coverage. Linking administrative hospital records with municipality-level environmental estimates allows the evaluation of spatial differences in asthma-coded admission rates across the country. However, because the present analysis is based on aggregated municipality-year data and does not include individual-level children without hospitalization, its findings should be interpreted as ecological associations between long-term ambient exposure and admission rates, not as individual-level causal risks.

In this study, “long-term exposure” is defined operationally as the annual mean ambient pollutant concentration assigned to each municipality and calendar year. The primary analysis did not estimate individual cumulative exposure before admission. The objective was to examine whether annual municipality-level air pollution was associated with asthma-coded respiratory hospital admission rates among children aged 0–3 years in Spain between 2020 and 2022. Secondary objectives were to compare rural and urban municipalities, formally test pollutant-by-urban interactions, and evaluate the robustness of particulate-matter estimates to zero-heavy counts, overdispersion, province fixed effects, residual spatial autocorrelation, and multipollutant collinearity. Because socioeconomic indicators were not available in the linked analysis file, residual area-level confounding was treated as a central interpretative limitation rather than assuming that the ecological models fully adjusted for area-level determinants of hospitalization.

## 2. Materials and Methods

### 2.1. Study Design

We conducted a nationwide ecological municipality-year study of asthma-coded hospital admissions in children aged 0–3 years in Spain between 2020 and 2022. Clinical and administrative data were obtained from the Spanish Minimum Basic Data Set/Registry of Specialized Care Activity (MBDS/RAE-CMBD), an administrative database provided by the Spanish Ministry of Health with high national hospital-discharge coverage [21]. The RAE-CMBD/MBDS data used in this study were provided by the Spanish Ministry of Health as anonymised administrative hospital-discharge microdata through the formal data request procedure [22]. The available variables included an anonymised internal linkage variable, sex, birth date, dates of hospital admission and discharge, ICU admission, postcode/municipality-level residence information, diagnoses, procedure codes, discharge outcomes, and information coded according to ICD-10-CM [23]. The research team did not receive names, national identity numbers, direct personal identifiers, clinical record numbers, full residential addresses, or any other information that would allow direct identification of individual patients.

All analyses were retrospective, observational, and performed on anonymised administrative data, with results reported only in aggregated form. Data were treated with complete confidentiality according to Spanish legislation. On this basis, individual informed consent and formal ethics approval were not required, because the dataset used by the authors consisted of anonymised administrative microdata and did not contain identifiable personal data. This interpretation is supported by Recital 26 of Regulation (EU) 2016/679, which states that data-protection principles do not apply to anonymous information where the data subject is not, or is no longer, identifiable, including for statistical or research purposes [24]. It is also consistent with the guidance of the Spanish Data Protection Agency, which states that anonymised data are not considered personal data and therefore are not governed by data-protection legislation [25], and with the Spanish data-protection framework for health research, including Organic Law 3/2018 [26].

The original hospital-discharge dataset included only children who experienced an asthma-coded hospital admission. Therefore, the study was not analyzed as an individual-level cohort of all children at risk. Instead, admissions were aggregated by municipality and year and analyzed as count data using the corresponding population of children aged 0–3 years as the denominator.

### 2.2. Study Variables and Outcomes

The outcome was defined as an asthma-coded respiratory hospital admission, operationalized as a hospital admission with a primary diagnosis coded as J45 occurring between 1 January 2020 and 31 December 2022. We use the term asthma-coded throughout the manuscript because, in children aged 0–3 years, a discharge code of J45 should not be interpreted as a validated diagnosis of persistent asthma. At these ages, severe recurrent wheeze, viral-induced wheeze, bronchiolitis-associated wheeze, transient early wheezing, and other preschool respiratory syndromes may overlap clinically with asthma-like hospital presentations [17]. The clinical descriptive analysis included the first asthma-coded hospitalization recorded for each child; repeated admissions for the same child were not counted as separate events in the descriptive or regression analyses. Children with prior admissions for selected respiratory infections were excluded according to predefined ICD-10-CM diagnosis codes available in the MBDS admission history. The available look-back period was limited to the hospital-discharge history available in MBDS; therefore, previous diagnoses treated exclusively outside hospital were not observable.

The primary ecological outcome was the annual number of asthma-coded hospital admissions among children aged 0–3 years in each municipality. This count was linked to the annual municipal population aged 0–3 years obtained from the Spanish National Statistics Institute (INE), which was used as the offset denominator in rate models [27]. Age- and sex-specific municipality-year denominators were not available in the analysis file; therefore, the primary denominator was the total population aged 0–3 years. This limitation is explicitly considered in the interpretation. In Spain, substantial differences exist between areas of high and low population density, with implications for traffic volumes and pollution mixtures [28]. Municipalities were classified into two geographic categories: urban areas, defined as those with a total population greater than 10,000 inhabitants, and rural areas, defined as those with fewer than 10,000 inhabitants [29]. This threshold was selected because it is transparent and reproducible in the available municipal population data; however, it is a crude simplification compared with population-density or DEGURBA-type classifications.

### 2.3. Air Pollution and Meteorological Data

Air pollutant data from 1 January 2020 to 31 December 2022 were obtained from the European Air Quality Forecasts of the Copernicus Atmosphere Monitoring Service (CAMS) [30]. CAMS provides hourly air quality analysis for the European Region at an approximate horizontal resolution of 10 km × 10 km. For each municipality-year, daily pollutant values were averaged over the full calendar year to obtain contemporaneous annual mean concentrations. These annual averages may include exposure time occurring after some admissions within the same year, and therefore should be interpreted as ecological annual exposure contrasts rather than individual pre-admission exposure histories. The pollutants examined were carbon monoxide (CO), nitrogen dioxide (NO_2_), sulfur dioxide (SO_2_), ozone (O_3_), PM_2.5_, and PM_10_, expressed in μg/m^3^.

Temperature and relative humidity were identified as the two main meteorological variables of interest. Data were acquired from the Copernicus Climate Change Service, specifically the ERA5-Land hourly dataset [31]. The ERA5-Land framework provides hourly analysis on a regular grid of (0.1°×0.1°). To estimate relative humidity, the daily average air temperature at 2 m above the ground surface was calculated, as well as the dew point temperature at the same height. These measures can be used to calculate relative humidity by [32]:

$$RH=100\times \left(\frac{\exp\left(\left(17.625\cdot T_{D}\right)/\left(243.04+T_{D}\right)\right)}{\exp\left((17.625\cdot T)/(243.04+T)\right)}\right)$$

where $T_{D}$ denotes the dew point temperature (°C) at 2 m above the surface, and *T* represents the air temperature (°C) at 2 m above the surface.

### 2.4. Linkage of Environmental and Health Data

Municipality-level air pollutant exposures were linked to health data using a high-resolution spatial interpolation framework. The municipality of residence was identified by retrieving the geographic coordinates of the municipality centroid. To assign environmental exposures at the municipal level, we applied the spatial extrapolation methodology described by Velez-Serrano and Alvaro-Meca [33]. Briefly, an adaptive spatial graph was constructed over all Spanish municipalities using Delaunay triangulation, with edge weights incorporating Euclidean distance, altitude differences, and population density factors. Pollutant and meteorological values from the CAMS and ERA5-Land grids were then interpolated onto each municipality centroid using an ensemble of barycentric interpolation and Gaussian Process Regression with a Matérn kernel (ν=1.5).

Admission-level accumulated exposure summaries were retained only for descriptive characterization of admitted children. The primary ecological regression analysis used contemporaneous annual municipality-level means merged with annual admission counts and child population denominators. Accordingly, all regression estimates refer to municipality-year exposure contrasts and not to individual exposure prior to admission.

### 2.5. Statistical Analysis

Categorical variables were described by absolute frequencies and percentages, while continuous variables were summarized using the median and interquartile range. To compare categorical variables by rural or urban setting, either the chi-square test or Fisher’s exact test was employed when appropriate. For continuous variables, the Kruskal–Wallis test was used.

The main regression analysis was conducted at the municipality-year level. Let $Y_{my}$ denote the number of asthma-coded hospital admissions among children aged 0–3 years in municipality *m* and year *y*, and let $N_{my}$ denote the corresponding child population. We fitted Poisson generalized linear models of the form:

$$Y_{my}∼\mathrm{Poisson}\left(\mu_{my}\right),$$

$$\log\left(\mu_{my}\right)=\log\left(N_{my}\right)+\alpha +\beta X_{my}+\gamma U_{m}+\delta_{y}+f_{1}\left(Temp_{my}\right)+f_{2}\left(Hum_{my}\right),$$

where $\log(N_{my})$ was included as an offset, $X_{my}$ represented the annual municipality-level pollutant exposure, $U_{m}$ indicated urban versus rural status, $\delta_{y}$ represented calendar-year fixed effects, and $f_{1}$ and $f_{2}$ were cubic spline terms for annual temperature and relative humidity. Pollutants were scaled by their interquartile range (IQR), so coefficients are presented as incidence rate ratios (IRRs) per IQR increase in each pollutant. Standard errors were clustered by municipality.

Because the outcome had many zero counts and the study period overlapped with the COVID-19 pandemic, several diagnostics and sensitivity analyses were conducted. First, crude admission rates per 100,000 children aged 0–3 years were tabulated overall and by rural–urban status for each calendar year, together with the proportion of municipality-years with zero admissions and the median/IQR of admissions. Second, we examined Pearson dispersion statistics and fitted negative-binomial models for PM_2.5_ and PM_10_ as overdispersion sensitivity analyses. Third, to evaluate whether the apparently stronger urban estimates reflected true effect modification, we fitted formal pollutant-by-urban interaction models. Fourth, to partially account for broad regional differences in coding, hospital access, admission practices, and residual spatial confounding, we fitted Poisson models with province fixed effects for the particulate-matter pollutants. Fifth, residual spatial autocorrelation was explored by calculating Moran’s I for Pearson residuals by calendar year using municipality-centroid k-nearest-neighbour weights. Municipality-level socioeconomic indicators, deprivation indices, education, income, unemployment, housing quality, and healthcare-access variables were not present in the linked analysis file, so a socioeconomic sensitivity analysis could not be performed. This limitation was therefore handled by explicitly restricting the causal interpretation and by discussing residual area-level confounding.

The main inferential focus was the particulate matter family, represented by PM_2.5_ and PM_10_. First, we fitted separate unipollutant models for PM_2.5_, PM_10_, NO_2_, SO_2_, O_3_, and CO to compare pollutant-specific associations under a common modeling framework. Second, we repeated the unipollutant models after stratification by rural and urban setting. Third, we fitted an exploratory multipollutant model including all six pollutants simultaneously and report this model only as an appendix sensitivity analysis because high collinearity made independent pollutant-specific interpretation unstable. Pairwise Spearman correlations and variance inflation factors (VIFs) were calculated to assess collinearity.

For visualization purposes, pollutant concentrations were grouped into predefined exposure categories to facilitate the interpretation of spatial maps. These categories were not used in the primary regression models, where pollutants were analyzed as continuous variables scaled by their interquartile range. Therefore, map categories should be interpreted as descriptive exposure intervals rather than as clinical, regulatory, or causal thresholds. All analyses were performed using Python version 3.13.2.

## 3. Results

Between 2020 and 2022, there were 3273 asthma-coded hospital admissions in children aged 0–3 years in the descriptive hospital-discharge dataset. Of these, 2502 occurred in urban areas and 771 in rural areas. The clinical profiles of admitted children were comparable between the two settings (Table 1). Forty percent of admissions involved girls. The median age was two years, and the median length of stay was three days. No deaths were associated with these admissions. However, 6.5% of children required ICU admission. The most common comorbidity was obesity, present in 0.5% of cases. The median cost per admission was 2439.4 € (Table 1).

Median exposure to air pollutants differed between rural and urban areas in the descriptive admission-level dataset (Table 2). Urban areas showed higher concentrations of all air pollutants analyzed, except for ozone, which was more elevated in rural areas. Because this table is admission-weighted, it overrepresents municipalities with more admissions and should not be interpreted as the exposure distribution in the full population at risk.

The full municipality-year regression panel provided a different and more appropriate description of the exposure contrast underlying the regression models (Table 3). For example, the overall unweighted median [IQR] PM_2.5_ concentration was 6.42 [5.92–7.54] μg/m^3^, whereas the population-weighted median was 8.27 [6.43–9.59] μg/m^3^, reflecting the greater contribution of more populated municipalities. Similar differences between unweighted and population-weighted summaries were observed for PM_10_, NO_2_, SO_2_, and CO.

**Figure A1:**
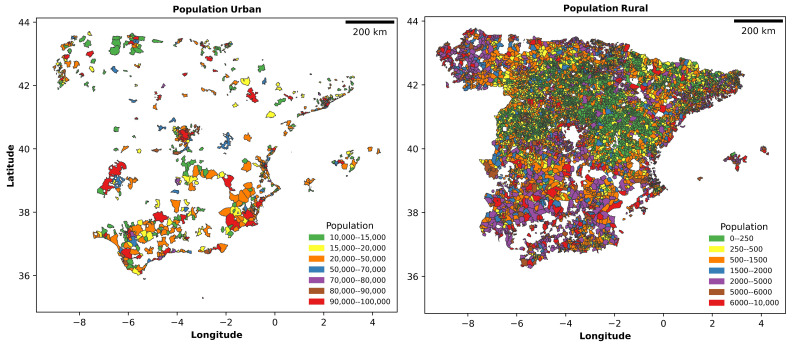
Distribution of municipal population by rural–urban geographical area.

**Figure A2:**
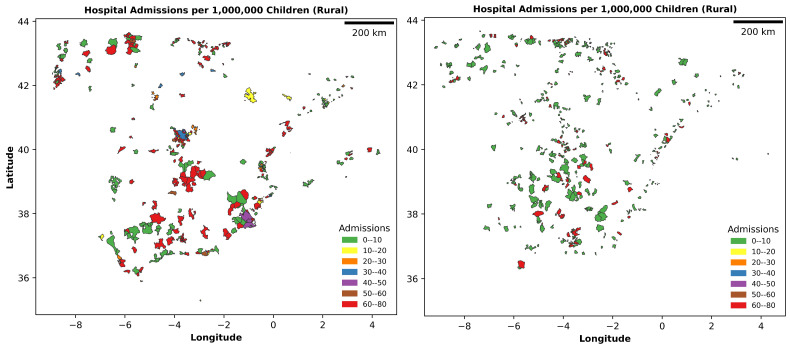
Spatial distribution of crude asthma-coded hospital admission rates by geographical area. Rates are based on first asthma-coded admissions included in the descriptive hospital-discharge dataset and are shown per 1,000,000 children aged 0–3 years.

**Figure A3:**
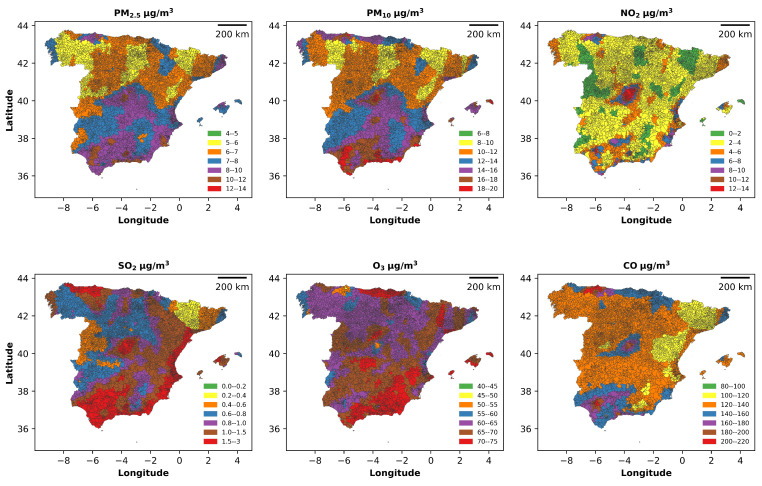
Spatial distribution of annual mean pollutant concentrations across Spanish municipalities.

**Figure A4:**
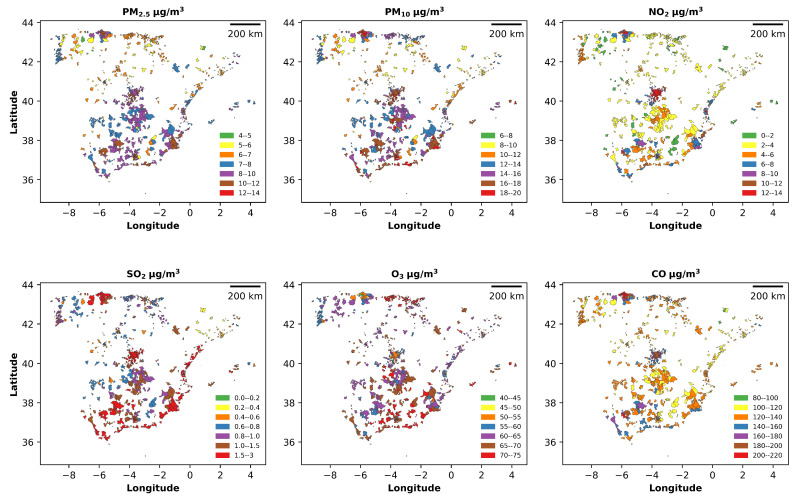
Spatial distribution of pollutant concentrations in municipalities with asthma-coded hospital admissions.

**Figure A5:**
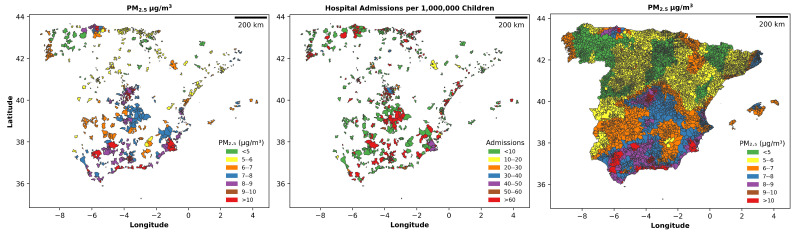
Distribution of asthma-coded hospital admissions and PM_2.5_. The right panel represents annual PM_2.5_ levels in all municipalities, the left panel represents PM_2.5_ levels in municipalities with hospital admissions, and the center panel represents crude first-admission rates per 1,000,000 children aged 0–3 years.

**Figure A6:**
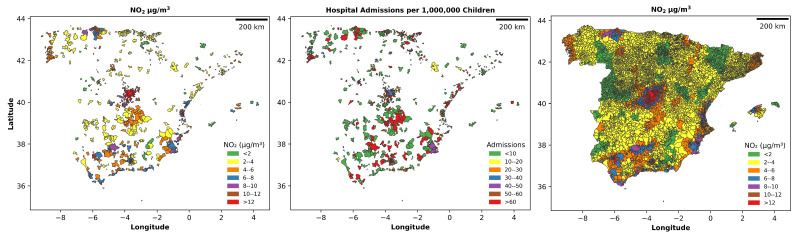
Distribution of asthma-coded hospital admissions and NO_2_. The right panel represents annual NO_2_ levels in all municipalities, the left panel represents NO_2_ levels in municipalities with hospital admissions, and the center panel represents crude first-admission rates per 1,000,000 children aged 0–3 years.

**Figure A7:**
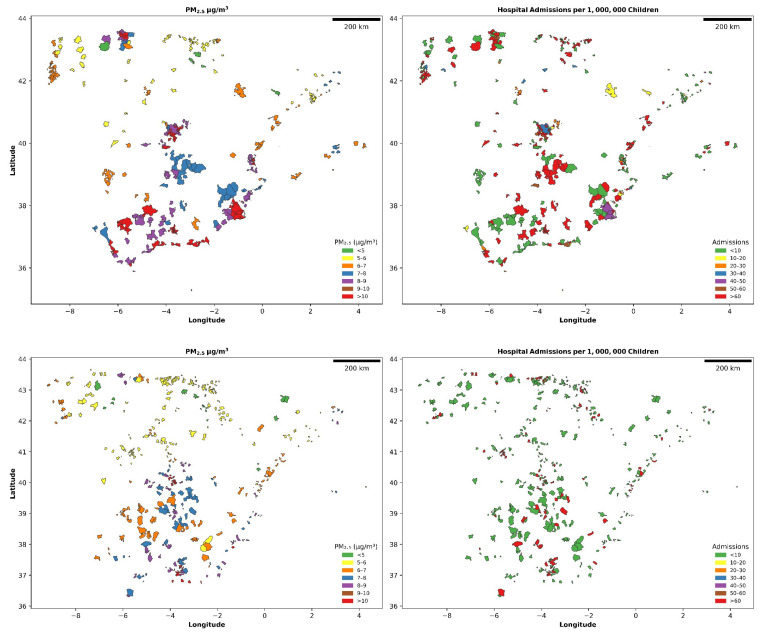
Distribution of asthma-coded hospital admissions and PM_2.5_ by urban (**top**) and rural (**bottom**) setting. Admission maps show crude first-admission rates per 1,000,000 children aged 0–3 years.

**Figure A8:**
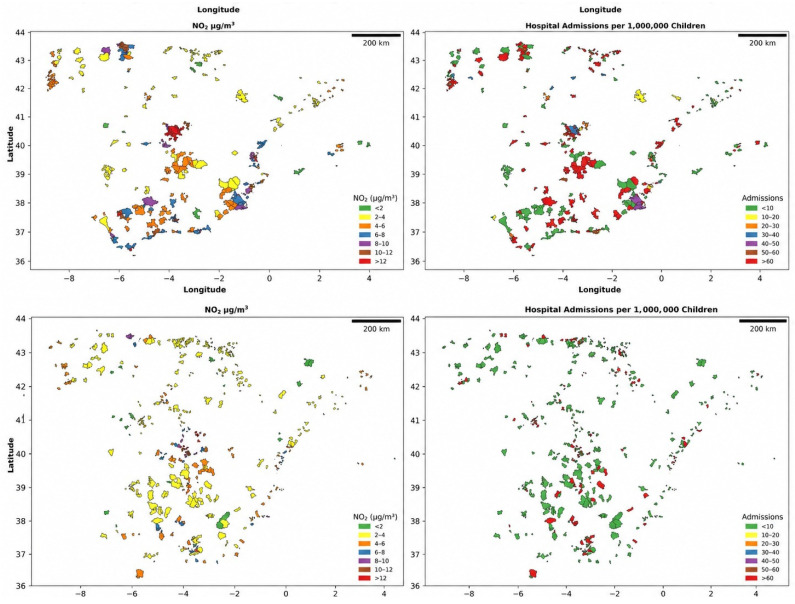
Distribution of asthma-coded hospital admissions and NO_2_ by urban (**top**) and rural (**bottom**) setting. Admission maps show crude first-admission rates per 1,000,000 children aged 0–3 years.

Spain is administratively divided into over 8000 municipalities, the majority of which are classified as rural, with fewer than 10,000 inhabitants (Figure A1). The spatial distribution of asthma-coded hospital admissions varied between urban and rural areas (Figure A2). Maps of pollutant concentrations showed distinct spatial patterns for PM_2.5_, PM_10_, NO_2_, SO_2_, O_3_, and CO in all municipalities (Figure A3) and in municipalities with asthma-coded admissions (Figure A4). For PM_2.5_ and NO_2_, areas with higher annual mean concentrations overlapped with several regions showing higher admission rates, particularly in urban and peri-urban areas (Figure A5 and Figure A6). The corresponding rural–urban maps for PM_2.5_ and NO_2_ are provided in Figure A7 and Figure A8. These maps are descriptive and should not be interpreted as individual-level exposure-response estimates.

After linkage to annual municipality-level child population denominators and environmental exposures, the ecological regression dataset included 20,746 municipality-year observations from 7251 municipalities and 3231 asthma-coded admissions. The difference between 3273 admissions in the descriptive dataset and 3231 admissions in the regression panel resulted from the construction of the annual ecological panel: 26 admissions in the source extraction had discharge dates outside the 2020–2022 analysis years, and 16 additional admissions were in municipality-years with a zero recorded child population denominator and were therefore excluded from rate modeling. The total child population represented in the regression panel was 5,454,406 municipality-year child observations.

Crude asthma-coded admission rates increased from 44.2 per 100,000 children aged 0–3 years in 2020 to 56.5 in 2021 and 78.5 in 2022 (Table 4). Urban rates increased from 40.4 to 75.9 per 100,000, whereas rural rates increased from 60.9 to 89.5 per 100,000. The outcome was sparse: overall, 93.7% of municipality-years had zero admissions and the median number of admissions per municipality-year was 0 [0–0]. In urban municipality-years, the zero-count proportion decreased from 68.8% in 2020 to 63.0% in 2022, whereas it remained above 96% in rural municipality-years.

In unipollutant

**Figure 1:**
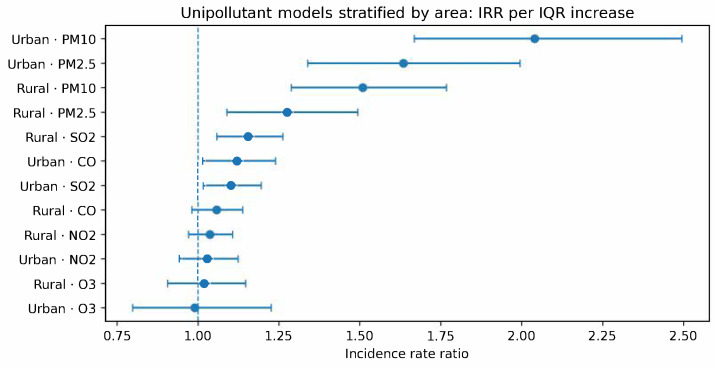
Ecological Poisson incidence rate ratios for asthma-coded hospital admission rates by pollutant and geographic setting. Pollutants were scaled by their interquartile range. Models were adjusted for calendar year, temperature, relative humidity, and, in the overall model, rural–urban status. Standard errors were clustered by municipality.

Poisson models adjusted for calendar year, urban–rural status, temperature, and relative humidity, the clearest and most consistent positive associations were observed for particulate matter (Figure 1, Table 5). Per IQR increase, the overall IRR was 1.54 (95% CI 1.28–1.85; p<0.001) for PM_2.5_ and 1.89 (95% CI 1.59–2.26; p<0.001) for PM_10_. SO_2_ showed a smaller positive association with admission rates (IRR 1.10, 95% CI 1.01–1.20; p=0.022). NO_2_, O_3_, and CO were not clearly associated with admission rates in the overall unipollutant models, although CO showed a borderline positive association (IRR 1.10, 95% CI 1.00–1.22; p=0.059).

When stratified by rural and urban setting, PM_2.5_ and PM_10_ remained positively associated with asthma-coded admission rates in both settings (Table 5). For PM_2.5_, the IRR per IQR increase was 1.28 (95% CI 1.09–1.49; p=0.002) in rural areas and 1.63 (95% CI 1.34–2.00; p<0.001) in urban areas. For PM_10_, the corresponding IRRs were 1.51 (95% CI 1.29–1.77; p<0.001) and 2.04 (95% CI 1.67–2.50; p<0.001), respectively. SO_2_ was also positively associated with admission rates in rural areas and urban areas. CO was positively associated only in urban areas.

Spearman correlations showed substantial collinearity between several pollutants, especially PM_2.5_ and PM_10_ (ρ=0.90), PM_2.5_ and NO_2_ (ρ=0.77), and NO_2_ and CO (ρ=0.65). The VIFs were 8.09 for PM_2.5_, 7.14 for PM_10_, and 4.10 for NO_2_. The full six-pollutant model is shown in Table A1 because the PM_2.5_ estimate reversed direction after adjustment for PM_10_, a pattern consistent with instability induced by collinearity rather than a biologically plausible protective effect. Accordingly, multipollutant results were interpreted only as evidence of collinearity within the pollutant mixture and were not used to isolate independent effects of PM_2.5_ versus PM_10_.

Formal interaction tests did not provide strong statistical evidence that particulate-matter associations differed by rural–urban status (Table A2). The urban-versus-rural ratio of IRRs was 1.06 (95% CI 0.86–1.31; interaction p=0.582) for PM_2.5_ and 1.22 (95% CI 0.97–1.54; interaction p=0.087) for PM_10_. Therefore, the larger urban point estimates are presented descriptively and should not be interpreted as definitive effect modification.

Model diagnostics supported the need for cautious interpretation. The Pearson dispersion statistic was 2.39 for the PM_2.5_ Poisson model and 2.61 for the PM_10_ Poisson model, indicating overdispersion. Negative-binomial sensitivity models attenuated but retained positive particulate-matter associations: IRR 1.46 (95% CI 1.36–1.57) for PM_2.5_ and 1.78 (95% CI 1.64–1.92) for PM_10_. Province fixed-effect Poisson models also attenuated the estimates but kept them positive: IRR 1.38 (95% CI 1.16–1.65) for PM_2.5_ and 1.30 (95% CI 1.17–1.44) for PM_10_. Detailed sensitivity estimates are reported in Table A3. Residual spatial autocorrelation was detectable in the main models by year-specific Moran’s I (approximately 0.03–0.09), whereas Moran’s I was close to zero after province fixed effects in the PM_2.5_ model, suggesting that broad regional adjustment reduced residual spatial clustering (Table A4).

## 4. Discussion

In this nationwide ecological analysis of Spanish municipality-year data, long-term annual municipality-level exposure to particulate air pollution was associated with higher asthma-coded respiratory hospital admission rates among children aged 0–3 years. The main finding was not that one particulate fraction could be cleanly separated from the other, but rather that the particulate matter mixture showed a consistent signal: both PM_2.5_ and PM_10_ were positively associated with admission rates in unipollutant models, and both associations were present in rural and urban strata. Although point estimates were larger in urban municipalities, formal interaction tests did not provide conclusive evidence of rural–urban effect modification. These results should be interpreted as municipality-level associations with severe asthma-coded respiratory morbidity, not as individual-level hazard ratios, confirmed incident asthma, or direct causal effects.

Outcome validity is therefore central to the interpretation of the study. In preschool children, especially at ages 0–3 years, asthma labels in administrative discharge data may include heterogeneous severe wheezing phenotypes rather than physician-confirmed persistent asthma. Non-differential outcome misclassification would generally be expected to reduce specificity and may dilute true associations, whereas differential coding or admission practices across municipalities, hospitals, years, or pandemic phases could bias estimates in less predictable directions. For this reason, the outcome is described as asthma-coded respiratory hospitalization and the results are framed as hospital-burden indicators for severe wheezing/asthma-like morbidity rather than as asthma incidence estimates.

The particulate-matter interpretation is important because PM_2.5_ and PM_10_ were strongly correlated in our data. The exploratory multipollutant model showed an unstable pattern when both particulate fractions were entered simultaneously: PM_10_ remained positively associated with admissions, whereas the PM_2.5_ estimate became inverse after mutual adjustment. This type of reversal is biologically difficult to interpret and is more consistent with multicollinearity and mutual adjustment within an overlapping pollutant mixture than with a protective effect of fine particles. Therefore, the most defensible conclusion is that asthma-coded admission rates were associated with a broader particulate-matter signal, while the present data do not allow robust separation of the independent contributions of fine and inhalable particulate fractions.

The stronger PM_10_ point estimate should not be interpreted as evidence that coarse or inhalable particles are intrinsically more toxic than fine particles. PM_10_ may act as a marker of a broader particulate mixture, including coarse resuspended dust, road dust, mineral particles, agricultural or construction-related particles, and transported particles, while also sharing a substantial fine-particle component with PM_2.5_. Differences in spatial contrast, exposure-measurement error, source composition, and collinearity can make the PM_10_ coefficient larger than the PM_2.5_ coefficient in an ecological model even when the underlying biological toxicity of fine particles remains important.

This interpretation is consistent with previous evidence linking particulate air pollution to pediatric asthma morbidity. Ambient PM can trigger oxidative stress, epithelial injury, airway inflammation, and immune activation, all of which may contribute to asthma exacerbation or asthma-like wheezing episodes in susceptible children [5,8,12,13]. Early childhood is a particularly vulnerable window because the respiratory and immune systems are still developing, and young children inhale more air relative to body weight than adults. Recent cohort evidence from the ECHO CREW consortium found that early-life exposure to PM_2.5_ and NO_2_ was associated with childhood asthma incidence [15]. Long-term pediatric exposure has also been linked to lung-growth and lung-function trajectories, which provides additional clinical relevance beyond acute admission counts [11]. Meta-analytic and burden-of-disease studies have also reported associations between PM_2.5_, asthma emergency visits, and childhood asthma burden [19,20,34]. Our study adds to this literature by focusing on severe asthma-coded admissions in children aged 0–3 years and by using nationwide Spanish administrative data linked to municipality-level exposure estimates.

The magnitude of the PM_2.5_ and PM_10_ IRRs was relatively large for IQR-scaled annual exposure contrasts. These estimates should therefore be interpreted cautiously. They may partly reflect residual area-level confounding, ecological bias, spatial clustering, heterogeneous admission practices, healthcare access, population-density differences, or instability in small-area count data with many zero admissions. The attenuation observed in negative-binomial and province fixed-effect sensitivity models supports the view that the direction of the particulate-matter signal is more robust than its exact magnitude.

The stronger particulate matter point estimates in urban areas may reflect a combination of higher combustion-related exposure, traffic density, population density, and healthcare access patterns. Urban municipalities accounted for most admissions and showed higher population-weighted concentrations of PM_2.5_, PM_10_, NO_2_, SO_2_, and CO in the full regression panel. However, the presence of positive PM_2.5_ and PM_10_ associations in rural areas suggests that particulate exposure is also relevant outside large urban settings. Rural particulate matter may reflect a different mixture of sources, including transported pollution, mineral dust, agricultural activity, biomass burning, domestic heating, and wildfire-related particles. Because our exposure assessment was municipality-level and source apportionment was not performed, these interpretations should be considered hypotheses for future work rather than direct evidence from the present analysis.

The findings for gaseous pollutants were less consistent. NO_2_ showed clear urban–rural differences in descriptive exposure levels, but it was not clearly associated with admission rates in the adjusted ecological unipollutant models. SO_2_ showed a smaller positive association, and CO was associated only in urban strata. These results do not imply that gaseous pollutants are irrelevant to pediatric respiratory health. Instead, they indicate that, within this ecological municipality-year framework and after adjustment for population denominators, calendar year, urbanicity, temperature, and humidity, the most robust pattern was observed for particulate matter.

The public-health implication is that reducing particulate air pollution remains relevant for pediatric respiratory protection. The 2021 WHO air quality guidelines recommend annual mean limits of 5 μg/m^3^ for PM_2.5_ and 15 μg/m^3^ for PM_10_ [7]. The revised European Union Ambient Air Quality Directive has moved 2030 standards closer to WHO recommendations, including a stricter annual PM_2.5_ limit of 10 μg/m^3^, although this remains above the WHO guideline [35]. Our ecological analysis does not estimate the individual causal benefit of achieving these thresholds, but it supports the epidemiological relevance of particulate matter as a population-level marker of pediatric respiratory hospital burden.

Our study is not without limitations. First, because the available individual-level MBDS dataset included only children with asthma-coded admissions, the analysis could not be performed as an individual cohort of all children at risk. The regression analysis was therefore ecological, using municipality-year admission counts and child population denominators. Consequently, the results should not be interpreted as individual-level causal risk estimates. Second, outcome misclassification is possible and may be substantial. Asthma diagnosis in children aged 0–3 years is clinically challenging, and some admissions coded as asthma may correspond to recurrent viral wheeze, bronchiolitis-associated wheeze, transient early wheezing, infection-related wheezing, or other preschool respiratory syndromes rather than confirmed asthma. The administrative discharge code identifies the reason recorded for hospitalization, not a standardized specialist-confirmed asthma phenotype. This limitation may affect both the magnitude and, if coding practices vary geographically or over time, the direction of associations. Third, we did not have individual-level or area-level data on several potential determinants of admission rates, including socioeconomic deprivation, parental education, household income, unemployment, housing quality, crowding, parental smoking, indoor air pollution, childcare attendance, vaccination status, viral circulation, healthcare access, distance to hospital, primary care availability, admission thresholds, regional coding practices, or other markers of healthcare-seeking behavior. These factors may be associated with both ambient pollution and hospitalization risk and therefore represent an important source of residual confounding. Municipality-level socioeconomic indicators were not available in the linked analysis file, so socioeconomic sensitivity analyses could not be performed. Province fixed effects partially addressed broad regional heterogeneity, but they are not a substitute for individual- or municipality-level socioeconomic adjustment. Fourth, exposure was assigned at the municipality level using municipality centroids and interpolated environmental surfaces. This ecological assignment may misclassify exposure, particularly in geographically large municipalities, municipalities with strong altitude gradients, coastal or mountain areas, and urban municipalities with marked within-municipality traffic or industrial contrasts. The approach also does not capture time-activity patterns, indoor exposures, residential history, individual mobility, or daycare/school locations. The exposure metric was the contemporaneous annual municipality-level mean, not a lagged annual average or individual pre-admission cumulative exposure. Lagged annual exposure windows could not be implemented with the available linked ecological panel and are a priority for future analyses. Fifth, age- and sex-specific municipality-year denominators were not available in the analysis file; therefore, rates were not age–sex-standardized within the 0–3-year group despite the predominance of boys among admitted children. Sixth, the study period overlapped entirely with the COVID-19 pandemic and its aftermath. Respiratory virus circulation, non-pharmaceutical interventions, mobility, air pollution levels, healthcare-seeking behavior, and hospital admission thresholds changed during 2020–2022. Non-pharmaceutical interventions implemented during 2020 substantially suppressed common respiratory viruses and reduced asthma-related healthcare utilization in young children; once restrictions were relaxed, asthma emergency department visits rebounded markedly, and in children younger than 5 years, post-lockdown visits overcorrected above pre-pandemic levels [36]. Calendar-year fixed effects adjust for broad annual shifts, but they cannot capture the complex timing of viral epidemics, changes in admission thresholds, healthcare access, or post-pandemic rebound phenomena. Therefore, the observed increase in crude admission rates from 2020 to 2022 should not be interpreted as a secular increase in confirmed asthma burden. Seventh, the outcome contained many zero municipality-year counts and showed overdispersion. Negative-binomial sensitivity analyses reduced dispersion and attenuated the particulate-matter estimates but did not remove the positive PM signal. Residual spatial autocorrelation was also present in the main models, although it was reduced by province fixed effects. Finally, the high correlation between pollutants limits interpretation of multipollutant models and prevents firm conclusions about the independent effects of PM_2.5_, PM_10_, and gaseous co-pollutants. The full six-pollutant model is therefore presented as an appendix sensitivity analysis rather than as a primary result (Table A1).

Our study also has several strengths. First, this is a nationwide study covering Spain and using a high-coverage administrative hospital discharge database. Second, distinguishing between rural and urban areas allowed us to explore spatial heterogeneity in the association between air pollution and pediatric respiratory hospital burden. Third, exposure estimates were assigned at the municipality level using a spatial interpolation approach based on Delaunay triangulation and Gaussian Process Regression [33], rather than relying only on a few monitoring stations. Fourth, the analysis uses explicit population denominators and Poisson count models, which are better aligned with the available data structure than an individual time-to-event approach. Finally, the inclusion of crude rate tables, full-panel exposure distributions, formal interaction tests, overdispersion sensitivity analyses, province fixed effects, residual spatial-autocorrelation checks, and explicit statements on unavailable socioeconomic information makes the ecological design and its robustness limitations more transparent.

## 5. Conclusions

In conclusion, contemporaneous annual municipality-level particulate air pollution was associated with higher asthma-coded hospital admission rates in children aged 0–3 years in Spain. PM_2.5_ and PM_10_ showed positive associations in unipollutant models, with larger urban point estimates but no conclusive formal evidence of rural–urban effect modification. Because the analysis is ecological, the outcome was sparse, particulate fractions were highly correlated, and the study period coincided with the COVID-19 pandemic, the findings are best interpreted as evidence of a particulate-matter signal associated with pediatric respiratory hospital burden, rather than as individual-level causal estimates or as proof of an independent effect of one particulate fraction. These findings support the relevance of particulate matter as a population-level marker of pediatric respiratory hospital burden and justify further studies using individual-level designs.

## Figures and Tables

**Table 1:** Summary of clinical and epidemiological characteristics of children with asthma-coded respiratory hospital admissions between 2020 and 2022 in Spain, stratified by geographical area. Values are expressed as number (%) for categorical variables and median (interquartile range) for quantitative variables. Abbreviations: LOS, Length of Stay in Days; CHF, Congestive Heart Failure.

		Overall	Rural	Urban	*p*-Value
N		3273	771	2502	
Gender	Female	1296 (39.6)	304 (39.4)	992 (39.6)	0.947
	Male	1977 (60.4)	467 (60.6)	1510 (60.4)	
Age		2.0 [1.0, 3.0]	2.0 [1.0, 3.0]	2.0 [1.0, 3.0]	0.480
LOS		3.0 [2.0, 4.0]	3.0 [2.0, 4.0]	3.0 [2.0, 4.0]	0.217
Year	2020	872 (26.6)	226 (29.3)	646 (25.8)	0.145
	2021	1029 (31.4)	238 (30.9)	791 (31.6)	
	2022	1372 (41.9)	307 (39.8)	1065 (42.6)	
Death		0 (0.0)	0 (0.0)	0 (0.0)	1.000
ICU Admission		213 (6.5)	50 (6.5)	163 (6.5)	1.000
ICU Days		2.0 [1.0, 4.0]	3.0 [1.2, 4.0]	2.0 [1.0, 3.5]	0.044
Charlson Index		1.0 [1.0, 1.0]	1.0 [1.0, 1.0]	1.0 [1.0, 1.0]	0.466
Obesity		16 (0.5)	4 (0.5)	12 (0.5)	1.000
CHF		3 (0.1)	1 (0.1)	2 (0.1)	0.553
Diabetes		3 (0.1)	0 (0.0)	3 (0.1)	1.000
Cancer		5 (0.2)	2 (0.3)	3 (0.1)	0.337
Cost (€)		2439.4 [2102.6, 3063.8]	2226.8 [2102.6, 3063.8]	2450.3 [2102.6, 3090.6]	0.586

**Table 2:** Admission-level, admission-weighted summary of air pollutant concentrations by geographical area among children with asthma-coded admissions. Values are expressed as median (interquartile range). These summaries describe the admitted children and should not be interpreted as the exposure distribution in the population at risk.

	Overall	Rural	Urban	*p*-Value
N	3273	771	2502	
PM_2.5_ (μg/m^3^)	8.1 [6.7, 9.5]	7.1 [6.1, 8.5]	8.5 [6.9, 9.8]	<0.001
PM_10_ (μg/m^3^)	15.2 [12.7, 17.8]	13.6 [10.9, 16.1]	15.7 [13.3, 18.2]	<0.001
NO_2_ (μg/m^3^)	5.1 [3.2, 10.2]	3.4 [2.5, 5.2]	6.1 [3.6, 12.6]	<0.001
SO_2_ (μg/m^3^)	1.6 [0.9, 2.6]	1.0 [0.7, 1.5]	1.9 [0.9, 3.0]	<0.001
O_3_ (μg/m^3^)	61.6 [55.1, 67.9]	64.1 [58.9, 69.6]	60.7 [54.2, 67.3]	<0.001
CO (μg/m^3^)	129.9 [117.2, 156.2]	121.7 [112.6, 135.0]	133.6 [119.4, 163.8]	<0.001
Temperature (°C)	15.3 [12.4, 18.3]	15.0 [11.7, 18.1]	15.5 [12.7, 18.4]	0.002
Relative Humidity %	70.8 [60.1, 79.6]	70.3 [60.6, 79.0]	71.1 [59.9, 79.7]	0.428

**Table 3:** Distribution of annual pollutant concentrations in the full municipality-year regression panel, shown as unweighted and population-weighted median [IQR], overall and by rural–urban status. Concentrations are expressed in μg/m^3^.

Area	Pollutant	Unweighted Median [IQR]	Population-Weighted Median [IQR]
Overall	PM_2.5_	6.42 [5.92–7.54]	8.27 [6.43–9.59]
Overall	PM_10_	11.23 [10.02–13.48]	14.52 [11.40–16.60]
Overall	NO_2_	2.60 [2.01–3.62]	4.72 [2.62–9.66]
Overall	SO_2_	0.75 [0.58–1.11]	1.39 [0.73–2.29]
Overall	O_3_	64.23 [62.11–67.37]	63.43 [58.58–66.61]
Overall	CO	119.87 [114.10–126.94]	130.40 [119.87–145.37]
Rural	PM_2.5_	6.37 [5.88–7.35]	7.15 [6.25–8.46]
Rural	PM_10_	11.05 [9.94–13.17]	12.83 [10.62–14.90]
Rural	NO_2_	2.56 [1.97–3.36]	3.22 [2.26–4.78]
Rural	SO_2_	0.74 [0.57–1.04]	0.93 [0.65–1.43]
Rural	O_3_	64.27 [62.19–67.29]	64.03 [61.43–68.04]
Rural	CO	119.33 [113.87–125.71]	124.39 [117.16–132.47]
Urban	PM_2.5_	7.92 [6.48–9.26]	8.63 [6.59–9.72]
Urban	PM_10_	13.85 [11.86–16.02]	15.07 [11.83–16.76]
Urban	NO_2_	4.24 [2.69–6.48]	5.36 [2.79–10.98]
Urban	SO_2_	1.25 [0.73–1.91]	1.63 [0.77–2.51]
Urban	O_3_	63.57 [59.84–68.01]	63.20 [57.42–66.08]
Urban	CO	129.04 [119.91–137.69]	131.84 [120.76–152.87]

**Table 4:** Crude asthma-coded hospital admission rates and zero-count diagnostics in the municipality-year regression panel by calendar year and rural–urban status. Rates are crude rates per 100,000 children aged 0–3 years using annual municipality-level child population denominators.

Year	Area	Admissions	Children 0–3	Rate/100,000	Zero Municipality-Years (%)	Admissions, Median [IQR]
2020	Overall	841	1,902,508	44.2	94.3	0 [0–0]
2020	Rural	216	354,844	60.9	97.2	0 [0–0]
2020	Urban	625	1,547,664	40.4	68.8	0 [0–1]
2021	Overall	1025	1,813,041	56.5	93.7	0 [0–0]
2021	Rural	234	345,479	67.7	97.0	0 [0–0]
2021	Urban	791	1,467,562	53.9	64.9	0 [0–1]
2022	Overall	1365	1,738,857	78.5	93.0	0 [0–0]
2022	Rural	300	335,178	89.5	96.4	0 [0–0]
2022	Urban	1065	1,403,679	75.9	63.0	0 [0–1]

**Table 5:** Unipollutant ecological Poisson models for asthma-coded hospital admission rates among children aged 0–3 years. Pollutants were scaled by their interquartile range (IQR). Values are incidence rate ratios (IRRs) with 95% confidence intervals and *p*-values. Models were adjusted for calendar year, temperature, relative humidity, and, in the overall model, urban–rural status. Standard errors were clustered by municipality.

Pollutant	IQR	Overall	Rural	Urban
PM_2.5_	1.62	1.54 (1.28–1.85); p<0.001	1.28 (1.09–1.49); p=0.002	1.63 (1.34–2.00); p<0.001
PM_10_	3.46	1.89 (1.59–2.26); p<0.001	1.51 (1.29–1.77); p<0.001	2.04 (1.67–2.50); p<0.001
NO_2_	1.61	1.02 (0.94–1.11); p=0.613	1.04 (0.97–1.11); p=0.283	1.03 (0.94–1.12); p=0.513
SO_2_	0.53	1.10 (1.01–1.20); p=0.022	1.16 (1.06–1.26); p=0.001	1.10 (1.02–1.20); p=0.018
O_3_	5.26	0.99 (0.84–1.18); p=0.942	1.02 (0.91–1.15); p=0.739	0.99 (0.80–1.23); p=0.925
CO	12.84	1.10 (1.00–1.22); p=0.059	1.06 (0.98–1.14); p=0.140	1.12 (1.01–1.24); p=0.024

## Data Availability

Aggregated results are contained in the article and Appendix A. The MBDS/RAE-CMBD is the property of the Spanish Ministry of Health, and environmental data are available from CAMS and ERA5-Land. Researchers may request access to the MBDS/RAE-CMBD from the Spanish Ministry of Health through the formal data request procedure and retrieve environmental data from the corresponding Copernicus services [22,30,31]. The authors are not allowed to redistribute the raw MBDS/RAE-CMBD microdata.

## Appendix A. Supplementary Tables and Figures

The appendix tables provide the exploratory multipollutant model, formal interaction tests, particulate-matter sensitivity analyses, and residual spatial-autocorrelation diagnostics referenced in the main text.

**Table A1:** Exploratory multipollutant ecological Poisson model including all pollutants simultaneously. Values are mutually adjusted incidence rate ratios (IRRs) with 95% confidence intervals. The model was adjusted for calendar year, urban–rural status, temperature, and relative humidity, with standard errors clustered by municipality. VIF: variance inflation factor.

Pollutant	IQR	Adjusted IRR (95% CI)	*p*-Value	VIF
PM_2.5_	1.62	0.53 (0.35–0.79)	0.002	8.09
PM_10_	3.46	4.19 (2.81–6.24)	<0.001	7.14
NO_2_	1.61	0.92 (0.83–1.01)	0.083	4.10
SO_2_	0.53	1.11 (1.01–1.22)	0.034	2.46
O_3_	5.26	0.70 (0.60–0.83)	<0.001	1.61
CO	12.84	0.86 (0.77–0.96)	0.008	2.45

**Table A2:** Formal interaction tests from ecological Poisson models including the pollutant, rural–urban status, and their product term. Pollutants were scaled by their interquartile range.

Pollutant	Urban-vs.-Rural Ratio of IRRs	95% CI	Interaction *p*-Value
PM_2.5_	1.06	0.86–1.31	0.582
PM_10_	1.22	0.97–1.54	0.087
NO_2_	0.97	0.89–1.07	0.552
SO_2_	0.96	0.85–1.08	0.507
O_3_	0.92	0.72–1.16	0.472
CO	1.07	0.95–1.20	0.286

**Table A3:** Sensitivity analyses for particulate-matter estimates. The province fixed-effect PM_10_ model used model-based standard errors because clustered covariance estimation was computationally unstable in the large fixed-effect model.

Pollutant	Model	IRR	95% CI	Pearson Dispersion
PM_2.5_	Main Poisson, clustered SE	1.54	1.28–1.85	2.39
PM_2.5_	Negative binomial	1.46	1.36–1.57	1.83
PM_2.5_	Poisson + province fixed effects	1.38	1.16–1.65	2.11
PM_10_	Main Poisson, clustered SE	1.89	1.59–2.26	2.61
PM_10_	Negative binomial	1.78	1.64–1.92	1.99
PM_10_	Poisson + province fixed effects	1.30	1.17–1.44	2.16

**Table A4:** Year-specific Moran’s I statistics for Pearson residuals from particulate-matter models. Spatial weights were based on municipality-centroid k-nearest neighbours (k = 5). The permutation *p*-values are two-sided and based on 19 permutations.

Pollutant	Model	Year	Moran’s I	Permutation *p*-Value
PM_2.5_	Main Poisson	2020	0.034	0.05
PM_2.5_	Main Poisson	2021	0.054	0.05
PM_2.5_	Main Poisson	2022	0.090	0.05
PM_2.5_	Poisson + province fixed effects	2020	0.006	0.35
PM_2.5_	Poisson + province fixed effects	2021	−0.000	1.00
PM_2.5_	Poisson + province fixed effects	2022	0.006	0.45
PM_10_	Main Poisson	2020	0.026	0.05
PM_10_	Main Poisson	2021	0.050	0.05
PM_10_	Main Poisson	2022	0.077	0.05
